# P-641. Distinct Cytokine Profiles in Severe Community-acquired Pneumonia and Metabolic Dysfunction-associated Steatotic Liver Disease

**DOI:** 10.1093/ofid/ofae631.838

**Published:** 2025-01-29

**Authors:** Branimir Gjurasin, Leona Radmanic Matotek, Mia Jelicic, Neven Papic

**Affiliations:** University Hospital for Infectious Diseases Zagreb, Zagreb, Grad Zagreb, Croatia; University hospital for infectious diseases Zagreb, Zagreb, Grad Zagreb, Croatia; University hospital for infectious diseases Zagreb, Zagreb, Grad Zagreb, Croatia; University Hospital for Infectious Diseases Zagreb, Zagreb, Grad Zagreb, Croatia

## Abstract

**Background:**

Metabolic dysfunction-associated steatotic liver disease (MASLD) has recently been identified as a risk factor for severe community-acquired pneumonia (sCAP) outcomes. While MASLD is associated with chronic systemic inflammation, cytokine responses in patients with sCAP and MASLD have not been characterized. The aim of this study was to analyze differences in concentrations and kinetics of cytokines in sCAP patients with and without MASLD.

**Methods:**

A prospective cohort pilot study included patients hospitalized with sCAP defined according to IDSA criteria. Upon admission, clinical and laboratory data were collected, and liver fibroelastography was performed to determine the presence of liver fibrosis and steatosis. Serum concentrations of 13 cytokines (IL-4, IL-2, CXCL10, IL-1β, TNF-α, CCL2, IL-17A, IL-6, IL-10, IFN-γ, IL-12p70, TGF-β1 and IL-8) were analyzed using a multiplex bead-based assay by flow cytometry at admission and on day 5 of hospitalization.

**Results:**

Thirty-eight patients (26 males, median age of 69, [IQR 61-79] years) with sCAP (SOFA 3 [IQR 2-4], PaO2/FiO2 186 [IQR 150-241]) were included. Of them, 18 (47.4%) had MASLD. There were no differences in age, sex and baseline disease severity between the groups. Patients with MASLD more frequently had components of metabolic syndrome. Etiology was identified in 66%, most commonly influenza (50%) and *S. pneumoniae* (18%). There were no differences in routine laboratory findings at admission, except for lactate, urea, AST, ALT and LDH which were higher in the MASLD group. While at admission we observed no differences in cytokine concentrations, on the 5th-day patients with MASLD had significantly higher concentrations of CXCL10, IL-10, TNF-α, and lower TGF- β1. Both groups had similar cytokine kinetics with significant decreases in CXCL10, CCL2, IL-10, IL-8 and IL-6. However, distinct temporal changes were observed for TGF-β1 and IL-2 which increased only in patients without MASLD.

**Conclusion:**

In conclusion, MASLD is associated with distinct cytokine profiles and kinetics in sCAP. A better understanding of cytokine changes could potentially help elucidate the complex and aberrant immune responses in MASLD patients during sCAP.

**Disclosures:**

**All Authors**: No reported disclosures

